# Recent cannabis exposure and acute nicotine effects: insights from randomized, double-blind, placebo-controlled intravenous nicotine studies

**DOI:** 10.1093/ijnp/pyag030

**Published:** 2026-05-26

**Authors:** Gabriel P A Costa, R Ross MacLean, Mehmet Sofuoglu, Joao P De Aquino

**Affiliations:** Department of Psychiatry, Yale University School of Medicine, New Haven, CT, United States; Clinical Neuroscience Research Unit (CNRU), Connecticut Mental Health Center (CMHC), New Haven, CT, United States; Department of Psychiatry, Yale University School of Medicine, New Haven, CT, United States; VA Connecticut Healthcare System, West Haven, CT, USA; Department of Psychiatry, Yale University School of Medicine, New Haven, CT, United States; Clinical Neuroscience Research Unit (CNRU), Connecticut Mental Health Center (CMHC), New Haven, CT, United States; VA Connecticut Healthcare System, West Haven, CT, USA; Department of Psychiatry, Yale University School of Medicine, New Haven, CT, United States; Clinical Neuroscience Research Unit (CNRU), Connecticut Mental Health Center (CMHC), New Haven, CT, United States; VA Connecticut Healthcare System, West Haven, CT, USA

**Keywords:** cannabis, nicotine, aversion, reward, drug reinforcement

## Abstract

**Objective:**

Cannabis–nicotine co-use is common and may affect cessation outcomes, yet whether recent cannabis exposure alters nicotine’s acute effects in humans remains unclear. We examined whether recent, non-daily cannabis exposure modulates the acute subjective effects of intravenous (IV) nicotine.

**Methods:**

We pooled data from 2 randomized, double-blind, placebo-controlled studies using IV nicotine to isolate nicotine’s pharmacodynamics in adults who smoke tobacco cigarettes (*N* = 60). Participants completed 3 sessions (placebo, 0.1 mg, 0.2 mg nicotine/70 kg). Recent cannabis exposure was defined a priori (past-30-day use with positive urine 11-nor-9-carboxy-delta-9-tetrahydrocannabinol vs no past-30-day use with negative screen). Primary outcomes were peak Drug Effects Questionnaire (DEQ) composites—stimulatory, pleasurable, aversive—within 10 minutes post-infusion. Secondary outcomes included nicotine self-administration behavior (proportion of nicotine choices) and cardiovascular responses (heart rate, blood pressure). Linear mixed-effects models included dose, cannabis exposure, sex, and FTND.

**Results:**

Nicotine increased all DEQ domains dose-dependently (*P* < .0001). Aversive effects showed a significant dose x cannabis exposure interaction (χ^2^ = 13.31, *P* = .001): participants with recent cannabis exposure reported greater aversive responses at 0.2 mg (Cohen’s d’ = 0.83), with minimal between-group differences at placebo/0.1 mg. Nicotine self-administration did not differ by cannabis exposure status, and no dose x cannabis exposure interactions were observed for cardiovascular responses.

**Conclusions:**

Recent, non-daily cannabis exposure is thus associated with selectively greater aversive responses to a clinically relevant IV nicotine dose, without differential cardiovascular reactivity or altered nicotine choice. These findings support a shift in the aversive limb of nicotine’s dose–response and inform mechanistic and clinical studies on how cannabis exposure shapes nicotine reinforcement and cessation outcomes.

**Clinical trial registration:**

NCT01495819, https://clinicaltrials.gov/study/NCT01495819.

**Significant outcomes**

1) Recent, non-daily cannabis exposure was associated with selectively amplified aversive responses to IV nicotine at a clinically relevant dose (0.2 mg/70 kg; Cohen’s d′ = 0.83), with a significant dose x cannabis exposure interaction.
2) Enhanced aversive sensitivity occurred without corresponding increases in nicotine self-administration behavior or differential cardiovascular reactivity, suggesting a shift in the reward–aversion balance rather than generalized sensitization.
3) Individuals with recent cannabis exposure exhibited lower nicotine dependence and daily cigarette consumption, consistent with a profile in which amplified aversion may constrain nicotine intake.

**Limitations**

1) Cannabis exposure was classified dichotomously via self-report and urine 11-nor-9-carboxy-delta-9-tetrahydrocannabinol without fine-grained characterization of use patterns, product types, THC potency, or THC:CBD ratios.
2) Cannabis status was not randomized, limiting causal inference about whether altered nicotine sensitivity precedes or follows cannabis exposure.
3) The modest sample size was powered for large effects, the observed non-significant trends in stimulatory and pleasurable responses require replication in larger cohorts.

## Introduction

Cannabis and nicotine are two of the most commonly co-used psychoactive substances worldwide,^1-7^ a convergence accelerated by the rapid liberalization of cannabis laws^8^ and the proliferation of novel nicotine delivery systems, paralleled by the diversification of cannabis product formulations (eg, edibles, concentrates, and oils with varying concentrations of cannabis constituents).^9^^,^^10^ In the United States, the prevalence of cannabis-nicotine co-use rose from 4.5% in 2002 to 6.4% in 2021.^11^ The relationship is bidirectional: individuals who use nicotine products are 2-3 times more likely to use cannabis, and individuals who use cannabis are almost twice as likely to smoke tobacco cigarettes compared with those who use either substance alone.^12^^,^^13^ As legal markets expand, co-use accounts for an estimated 1 in 3 adult cannabis product consumers^14^ and 1 in 5 nicotine product consumers.^15^^,^^16^ The health burden of this growing population of individuals who co-use cannabis and nicotine has yet to be fully quantified.^17^

The clinical ramifications of cannabis-nicotine co-use extend beyond additive effects, as emerging evidence suggests synergistic interactions that may complicate cessation efforts and amplify health risks, including elevated carbon monoxide and carcinogen levels, and may compound respiratory symptoms such as chronic bronchitis.^18^^,^^19^ Longitudinal cohorts have demonstrated lower quit rates for both cannabis and tobacco relative to those who use either substance alone, even after controlling for dependence severity.^20^^,^^21^ In the International Tobacco Control (ITC) Four-Country Study, increasing cannabis frequency predicted a 48% reduction in 24-month tobacco-cessation success.^21^ Conversely, cannabis cessation attempts are associated with transient spikes in tobacco cigarette consumption, suggesting a drug-substitution dynamic that undermines dual-quit efforts.^16^ These findings underscore the need to elucidate the neurobiological mechanisms underlying the reinforcing properties of co-use,^22^ particularly given the substantial population-level harms that remain inadequately characterized.^17^

Preclinical studies have established the neurobiological basis for cannabis-nicotine interactions, which involves crosstalk between the endocannabinoid and cholinergic systems within mesolimbic reward circuits.^23-25^ Cannabinoid receptors 1 (CB1R) and nicotinic acetylcholine receptors (nAChRs) co-localize in the ventral tegmental area, nucleus accumbens, hippocampus, and amygdala.^23^^,^^24^ Mechanistic studies show that chronic exposure to delta-9-tetrahydrocannabinol (THC), the main psychoactive constituent of cannabis, lowers the nicotine dose required to establish reward—an effect blocked by CB1 antagonism.^26^ Reciprocally, nicotine pre-exposure enhances THC-evoked dopamine release.^26^ Repeated nicotine also induces cross-tolerance to THC-induced ataxia, via cerebellar nitric-oxide signaling.^27^ Pharmacological manipulation of the endocannabinoid system with neutral CB1R antagonists and inverse agonists attenuates nicotine self-administration and cue-induced reinstatement across species,^25^^,^^28^ underscoring CB1–nAChR crosstalk as a mechanistic lynchpin.

However, this crosstalk likely operates differently across nicotine’s rewarding vs. aversive effects. Nicotine exhibits an inverted-U reinforcing function in which higher doses engage aversive processes (eg, nausea, anxiety) that reduce intake. Convergent evidence implicates the medial habenula–interpeduncular nucleus (MHb–IPN) pathway in mediating this aversion.^29^ Distinct nicotinic receptor subtypes drive these opposing effects: α4β2 receptors primarily facilitate reward through mesolimbic dopamine release,^30^^,^^31^ while α5-containing receptors in the MHb-IPN mediate aversion and limit intake at higher doses.^32^^,^^33^ Because CB1R are expressed presynaptically within both reward and aversion circuits and regulate aversive learning,^34^ cannabinoid tone may differentially modulate the balance between nicotine-evoked reward and aversion.^34^ Notably, while exogenous cannabinoids may counteract some of the nicotine-induced aversive effects^29^^,^^35^ through their anxiolytic^36^ and antiemetic^37^ properties, it is unclear if these interactions contribute to high rates of co-use. Alternatively, it has been suggested that cannabis exposure could shift nicotine’s dose–response curve by preferentially sensitizing aversion-encoding circuits, given the dense expression of CB1R within the MHb–IPN pathway, which primarily mediates the aversive effects of nicotine.^38^^,^^39^

Despite this extensive epidemiological and mechanistic work showing bidirectional risk relationships between cannabis and tobacco use, critical gaps remain in our understanding of how cannabis exposure modulates nicotine’s acute pharmacodynamic effects in humans. Previous human laboratory studies have yielded conflicting results, with transdermal nicotine enhancing subjective responses to smoked cannabis,^40^ while acute cannabis administration failed to alter tobacco smoking topography.^41^ Crucially, laboratory studies could not isolate nicotine’s pharmacodynamics from tobacco constituents and have not considered cannabis exposure status. By using intravenous (IV) nicotine, we directly test whether recent non-daily cannabis exposure alters acute nicotine effects.

We leveraged pooled data from 2 randomized, double-blind, placebo-controlled IV nicotine studies^42^^,^^43^ to test the hypothesis that recent cannabis exposure potentiates nicotine-induced stimulation and pleasure, while amplifying aversive effects at higher doses—a pattern consistent with CB1R modulation of nicotine-induced reward and aversion. We additionally examined whether cannabis exposure modulated nicotine self-administration behavior and cardiovascular responses. These interactions have direct clinical relevance: if cannabis exposure alters nicotine’s pharmacodynamic profile, first-line cessation pharmacotherapies (eg, nicotine replacement therapy dosing, varenicline) may require tailoring for the growing population of individuals who co-use cannabis and nicotine, especially as higher cannabis use frequency is linked to lower cessation success.^21^

## Materials and methods

### Participants

Here we report on a secondary analysis of pooled data. We combined data from 2 previously published studies examining IV nicotine self-administration in individuals who smoke tobacco cigarettes (NCT01495819^42^^,^^43^). To ensure comparability, we selected data from test sessions administering the same nicotine doses across the 2 studies: placebo (saline), 0.1 mg, and 0.2 mg/70 kg body weight. The combined sample comprised 60 participants (Supplementary Figure S1), evenly divided between individuals with and without recent cannabis exposure.

Study 1 (Jensen et al.; *n* = 26) enrolled nicotine-dependent individuals who smoked tobacco cigarettes regularly, with no upper age limit. Study 2 (MacLean et al.; *n* = 34) recruited non-dependent individuals who smoked tobacco between the ages of 18 and 30 years, smoked 5 or fewer tobacco cigarettes per day (CPD), and had Fagerström Test for Nicotine Dependence (FTND) scores less than 3. All participants were required to have smoked tobacco for at least 1 year and reported lifetime consumption of at least 100 tobacco cigarettes. Participants were not seeking treatment for nicotine dependence at the time of enrollment.

Exclusion criteria included current substance use disorders (except nicotine/cannabis), positive drug screens (except cannabis), abnormal physical/laboratory examinations, interfering psychiatric/medical conditions, and pregnancy/nursing.

All participants provided written informed consent prior to study participation. The studies were approved by the Institutional Review Boards of Yale University and the VA Connecticut Healthcare System.

### Study design and procedures

Both studies employed double-blind, placebo-controlled, crossover designs and equivalent experimental procedures. The sequence of nicotine dose assignments was counterbalanced to minimize the order effect. Participants completed multiple experimental sessions, each separated by 2-7 days to mitigate potential carryover effects.

Prior to each experimental session, participants were instructed to abstain from tobacco smoking after 10:00 pm the previous evening. Compliance with overnight abstinence was verified by expired carbon monoxide levels of 10 parts per million or less (8 ppm in Study 2) measured using a BreathCO monitor (Vitalograph, Inc., Lenexa, KS). Blood samples were collected at baseline to quantify nicotine and cotinine levels.

All sessions were conducted in the morning (approximately 08:00 am). Upon arrival, an indwelling IV catheter was inserted into the participant’s antecubital vein for nicotine administration and blood sampling. Cardiac rhythm was monitored continuously during infusions.

### Nicotine administration

The studies examined 3 nicotine dose conditions: placebo (0.9% saline), 0.1 mg nicotine/70 kg body weight, and 0.2 mg nicotine/70 kg body weight, with maximum nicotine doses capped at 0.1 and 0.2 mg, respectively. These doses were selected to approximate the nicotine delivery from 1 to 2 puffs of a typical tobacco cigarette. Based on participant body weights, the actual total nicotine administered at the 0.2 mg/70 kg dose ranged from approximately 0.13 to 0.2 mg, approximating the nicotine delivery from 1 to 3 puffs of a typical tobacco cigarette. Nicotine solutions were prepared by the research pharmacy using nicotine bitartrate dihydrate dissolved in 0.9% sodium chloride, adjusted for molecular weight to reflect nicotine free base.

### Measures

#### Baseline characteristics

Demographic information and smoking history were assessed at screening. Key baseline measures included CPD, the FTND, the Brief Questionnaire of Smoking Urges (BQSU), and the Positive and Negative Affect Schedule (PANAS).

Participants who used cannabis daily or who met the DSM-5 criteria for cannabis use disorder were excluded from study participation. These exclusions were part of the parent study safety protocols for IV nicotine administration. Daily cannabis use was additionally excluded to prevent acute intoxication or withdrawal from confounding nicotine response measurements. Among those who were enrolled, cannabis exposure status was operationalized a priori: individuals who reported cannabis use within the past 30 days and tested positive for cannabinoids on an 11-nor-9-carboxy-THC (THC-COOH) urine screen. THC-COOH, the primary inactive metabolite of THC, remains detectable in urine substantially longer than parent THC—typically 2-3 days after occasional use and up to 30 days following regular consumption at the standard cutoff of 50 ng/mL.^44^^,^^45^ This extended detection window enables reliable retrospective confirmation of recent cannabis exposure. Individuals categorized as unexposed to cannabis had denied using cannabis in the past 30 days and had a negative THC-COOH screen. In both studies, a urine drug screen was administered before each session to assess for recent use of opiates, phencyclidine, cocaine, amphetamines, and benzodiazepines. Participants with positive results (excluding cannabis) were excluded from that session.

#### Subjective drug effects

The primary outcome measure was the Drug Effects Questionnaire (DEQ), administered before and at 1, 3, 5, 8, and 10 minutes after the IV nicotine infusion. The DEQ consists of 9 items rated on 100-mm visual analog scales anchored from “not at all” (0) to “extremely” (100). Based on prior factor analyses demonstrating high intercorrelations,^42^^,^^43^ items were grouped into 3 composite scores: Stimulatory Composite (average of “feel stimulated,” “feel drug strength,” and “feel high”), Pleasurable Composite (average of “like drug effects”, “feel good,” and “want more”), and Aversive Composite (average of “feel anxious,” “feel down,” and “feel bad drug effects”).^46^ Peak values across all post-infusion time points were extracted for analysis.

#### Nicotine self-administration

Participants completed a forced-choice self-administration procedure. After sampling both the nicotine dose and placebo during the initial assessment period, participants were given multiple opportunities to choose between the 2 infusions, which remained labeled as “A” or “B” throughout the session. Each choice trial was separated by 15 minutes, and upon selection, the chosen infusion was immediately administered over 30 seconds via infusion pump. Due to differences in the original study protocols, the number of choice trials varied by study (6 ^42^ and 10 ^43^ choices per session). The dependent variable for self-administration was the proportion of nicotine choices (number of times nicotine was chosen divided by total number of choice trials).

#### Cardiovascular effects

Heart rate and blood pressure were measured immediately before and at 1, 2, 3, and 5 minutes after each infusion using automated monitors. Peak values were calculated for analysis.

### Statistical analysis

All analyses were conducted using R statistical software version 4.3.3.^47^ Continuous baseline variables were compared between participants with and without cannabis exposure using independent samples t-tests or Wilcoxon rank-sum tests as appropriate, while categorical variables were examined using chi-squared tests or Fisher’s exact tests. Statistical significance was set at *P* < .05.

For the primary analyses of DEQ composite scores, linear mixed-effects models (LMM) were employed. Due to positive skew in the subjective ratings, outcomes were log-transformed. The primary model specification included fixed effects for nicotine dose (placebo, 0.1 mg, 0.2 mg), cannabis exposure status (exposed vs unexposed), their interaction, sex, and FTND score as covariates. To account for within-subject correlations across doses, participant was included as a random intercept. For cardiovascular outcomes (systolic blood pressure, diastolic blood pressure, and heart rate), similar LMM were employed.

For nicotine self-administration, a generalized LMM with binomial distribution was employed to model the number of nicotine choices out of the total available choices (6 or 10, depending on study paradigm). Odds ratios (ORs) comparing individuals with cannabis exposure to those without within each dose level were calculated using estimated marginal means. In Study 1, all 26 participants completed all 3 dose conditions. In Study 2, 29 of 34 participants completed all 3 dose conditions, 3 were missing the 0.1 mg/70 kg session, 1 was missing the 0.2 mg/70 kg session, and 1 completed only the placebo session. The self-administration analysis additionally required usable forced-choice data and therefore included 58 of 60 participants (all 26 from Study 1 and 32 of 34 from Study 2); among the Study 2 participants, 28 contributed both active doses, 3 only the 0.2 mg/70 kg dose, and 1 only the 0.1 mg/70 kg dose. Linear mixed-effects models accommodate such unbalanced data by estimating parameters from all available observations under a missing-at-random assumption, without requiring listwise deletion or imputation.^48^ Individual DEQ item analyses are reported in Supplementary Table S6. Sensitivity analyses evaluating alternative covariate specifications are reported in Supplementary Tables S1–S5.

Due to observed group differences in nicotine dependence severity between cannabis-exposure groups (cannabis-exposed: 2.3 ± 2.3 vs unexposed: 3.9 ± 3.0, *P* = .028), model selection included FTND as a covariate. Sex was included as a covariate given convergent evidence showing the influence of sex on nicotine responses.^42^ Post-hoc pairwise comparisons between cannabis exposure groups (exposed vs unexposed) at each dose level were conducted using estimated marginal means. To quantify the magnitude of group differences, we report Cohen’s d’ effect sizes, which were calculated as the difference between group estimated marginal means divided by the pooled residual standard deviation from the mixed-effects model.

## Results

### Participant characteristics

The sample consisted of 60 participants, among whom 30 had recent cannabis exposure and 30 did not. Groups were well-matched demographically, with the sample being predominantly male (58%) and Black (68%), with a mean age of 31.5 years. Participants with cannabis exposure were slightly younger than those without (28.9 vs 34.0 years, *P* = .011).

Participants with cannabis exposure smoked approximately 40% fewer tobacco CPD than those without (8.2 vs 13.4 cigarettes, *P* = .046), and showed significantly lower nicotine dependence scores (FTND: 2.3 vs 3.9, *P* = .028). Participants with cannabis exposure also reported lower tobacco smoking urges, particularly for the desire and intention to smoke tobacco. Detailed participant characteristics are presented in Table 1.

**Table 1:** Participant demographics and smoking characteristics by cannabis exposure status.

Characteristic	No cannabis exposure	Cannabis exposure	Overall	
	*N* = 30^*a*^	*N* = 30^*a*^	*N* = 60^*a*^	*P*-value^*b*^
Age (years)	34.0 (8.8)	28.9 (5.9)	31.5 (7.9)	**.011**
Sex				.295
Male	15 (50%)	20 (67%)	35 (58%)	
Female	15 (50%)	10 (33%)	25 (42%)	
Race				>.999
Black	20 (67%)	21 (70%)	41 (68%)	
White	5 (17%)	5 (17%)	10 (17%)	
Hispanic	5 (17%)	4 (13%)	9 (15%)	
Weight (kg)	87.3 (21.5)	74.9 (18.7)	81.3 (21.0)	**.026**
Cigarettes per day	13.4 (11.0)	8.2 (8.6)	10.8 (10.1)	**.046**
FTND score	3.9 (3.0)	2.3 (2.3)	3.1 (2.8)	**.028**
BQSU total score	42.8 (16.6)	35.3 (16.4)	39.1 (16.8)	.083
BQSU factor 1	26.5 (9.7)	21.6 (9.9)	24.0 (10.1)	.055
BQSU factor 2	16.3 (8.5)	13.7 (7.5)	15.0 (8.1)	.226
PANAS positive	32.9 (8.5)	31.2 (8.4)	32.0 (8.4)	.431
PANAS negative	13.7 (5.3)	13.7 (4.1)	13.7 (4.7)	.978

^a^ *Mean (SD); n (%)*

^b^ *Wilcoxon rank sum test; Pearson’s Chi-squared test; Fisher’s exact test*. Boldface indicates statistically significant between-group differences (*P* < .05).

### Subjective effects of IV nicotine

#### Stimulatory effects

**Figure 1:**
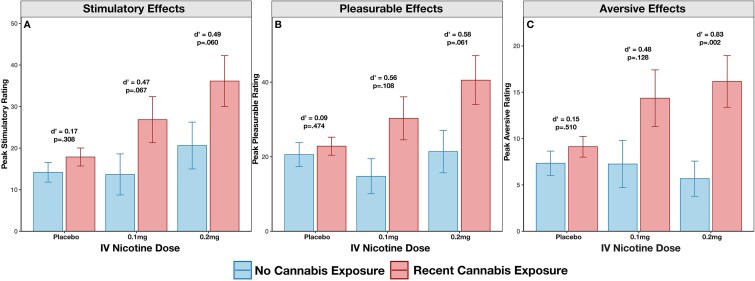
Subjective effects of intravenous nicotine by cannabis exposure status. Peak ratings for (A) stimulatory, (B) pleasurable, and (C) aversive effects following IV nicotine administration (placebo, 0.1 mg, 0.2 mg) in individuals with recent cannabis exposure (*n* = 30) and individuals without (*n* = 30). Values represent mean ± SEM on 100-point visual analog scales. Cohen’s d’ effect sizes and *P*-values from post-hoc comparisons between cannabis exposure groups are shown for each dose. Individuals with recent cannabis exposure reported consistently higher stimulatory and pleasurable effects across doses, though differences reached statistical significance only for aversive effects at the 0.2 mg dose (d’ = 0.83, *P* = .002). The significant dose x cannabis exposure interaction for aversive effects (*P* = .001) reflects the divergent dose–response patterns between groups, with individuals with recent cannabis exposure showing greater sensitivity to the aversive effects of nicotine at higher doses.

Both nicotine doses produced robust, dose-dependent increases in stimulatory effects compared to placebo (main effect of dose: χ^2^(2) = 50.74, *P* < .0001, Figure 1).

Participants with cannabis exposure reported stronger stimulation across all nicotine doses, with the main effect of cannabis exposure status approaching statistical significance (χ^2^(1) = 3.58, *P* = .058). The magnitude of group differences increased from small at placebo (d’ = 0.17, *P* = .308) to moderate at both active nicotine doses: 0.1 mg (d’ = 0.47, *P* = .067) and 0.2 mg (d’ = 0.49, *P* = .060).

As expected, higher nicotine dependence, indexed by FTND scores, was associated with blunted stimulatory responses to acute nicotine administration (β = -0.208, *P* = .0009).

#### Pleasurable effects

The pattern for pleasurable effects paralleled stimulatory responses, with a significant main effect of nicotine dose (χ^2^(2) = 55.91, *P* < .0001). Participants with cannabis exposure reported higher pleasurable ratings following nicotine administration, though the main effect of cannabis exposure status did not reach significance (χ^2^(1) = 2.62, *P* = .105). Effect sizes ranged from negligible at placebo (d’ = 0.09, *P* = .474) to moderate at both active doses: 0.1 mg (d’ = 0.56, *P* = .108) and 0.2 mg (d’ = 0.58, *P* = .061).

As with stimulatory effects, higher FTND scores predicted attenuated pleasurable responses (β = -0.224, *P* = .0008).

#### Aversive effects

For the aversive effects of nicotine, we observed significant main effects of nicotine dose (χ^2^(2) = 35.70, *P* < .0001) and cannabis exposure status (χ^2^(1) = 4.39, *P* = .036), as well as a significant dose x cannabis exposure status interaction (χ^2^(2) = 13.31, *P* = .001). Post-hoc analyses revealed minimally higher aversive effects for participants with cannabis exposure (vs unexposed) at placebo nicotine (d’ = 0.15, *P* = .510), which increased at 0.1 mg nicotine (d’ = 0.48, *P* = .128) and became pronounced at 0.2 mg nicotine (d’ = 0.83, *P* = .002). Consistent with the pattern observed for stimulatory and pleasurable effects, higher FTND scores predicted significantly attenuated aversive responses to nicotine (β = -0.117, *P* = .039). Individual item analyses revealed that this interaction was driven most strongly by “feeling down” and “bad drug effects” (Supplementary Table S6). Male participants reported higher aversive effects to nicotine than females (χ^2^(1) = 4.56, *P* = .033).

### Nicotine self-administration

**Figure 2:**
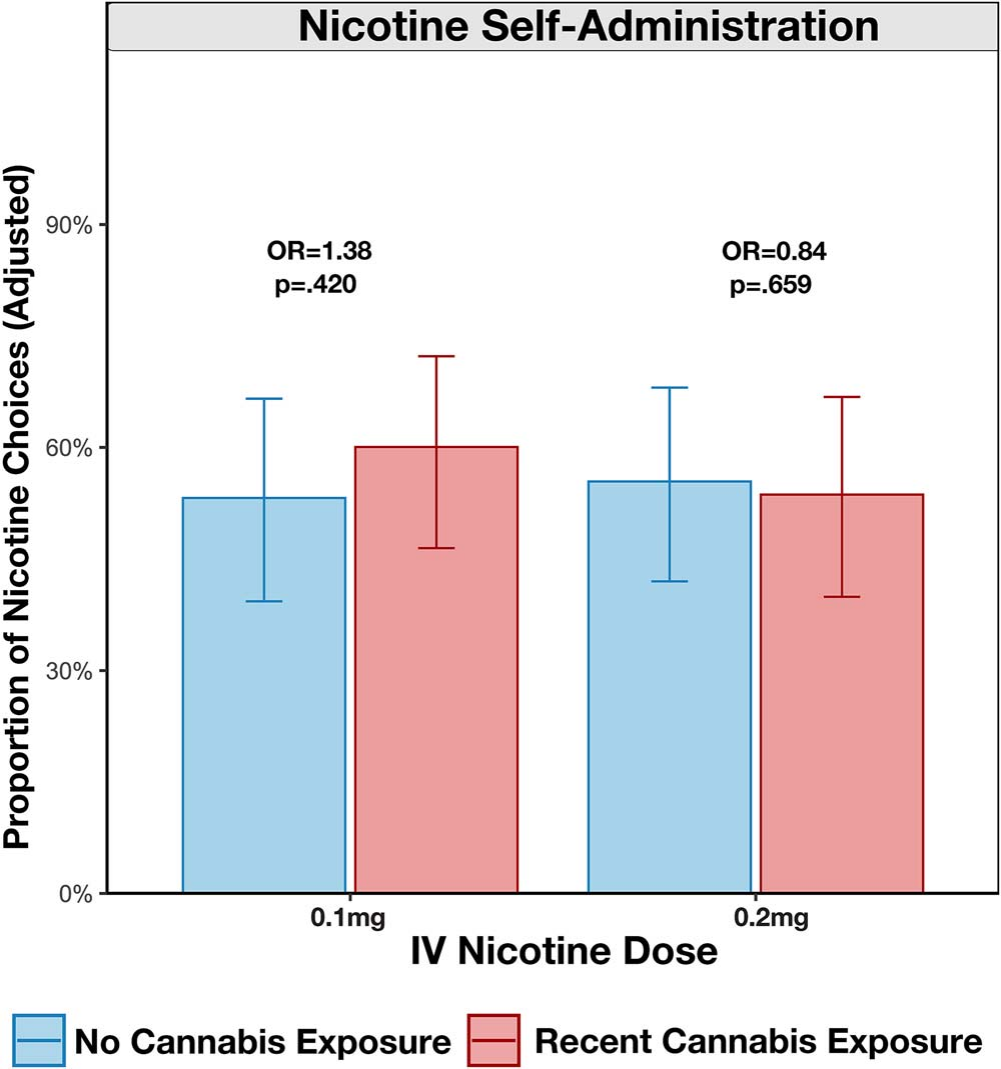
Nicotine self-administration by cannabis exposure status. Proportion of nicotine choices (adjusted) at 2 IV nicotine doses (0.1 mg, 0.2 mg) in individuals with recent cannabis exposure (*n* = 30) and individuals without (*n* = 30). Values represent estimated marginal means ±95% CIs from generalized linear mixed-effects models, adjusted for sex and FTND score. Odds ratios (OR) and *P*-values from post-hoc comparisons between cannabis exposure groups are shown for each dose. Neither group demonstrated a significant preference for nicotine over saline at either dose level, as confidence intervals included 50% (no preference). No significant main effect of cannabis exposure status (*P* = .812) or dose x cannabis exposure interaction (*P* = .319) was observed, indicating that enhanced subjective responses in individuals with recent cannabis exposure did not translate into increased nicotine self-administration behavior.

We found no significant main effect of cannabis exposure status on nicotine choice (*P* = .812). The cannabis exposure x dose interaction was also non-significant (*P* = .319), indicating that the relationship between nicotine dose and self-administration behavior did not differ between individuals with and without cannabis exposure (Figure 2).

At the 0.1 mg dose, individuals with cannabis exposure selected nicotine on an average of 58.6% of choice trials (95% CI, 45.1%, 70.9%) compared to 50.5% (95% CI, 37.0%, 63.9%) for those without (OR = 1.38, *P* = .420). At the 0.2 mg dose, individuals with cannabis exposure selected nicotine on 49.7% of trials (95% CI, 35.9%, 63.5%) compared to 54.1% (95% CI, 41.1%, 66.5%) for those without (OR = 0.84, *P* = .659). Neither group demonstrated a significant preference for nicotine over saline at either dose.

There was no significant main effect of sex (*P* = .686) or sex x cannabis exposure interaction (*P* = .571) on nicotine self-administration. FTND score was not a significant predictor of nicotine choice in the adjusted model (*P* = .339).

### Cardiovascular effects

**Figure 3:**
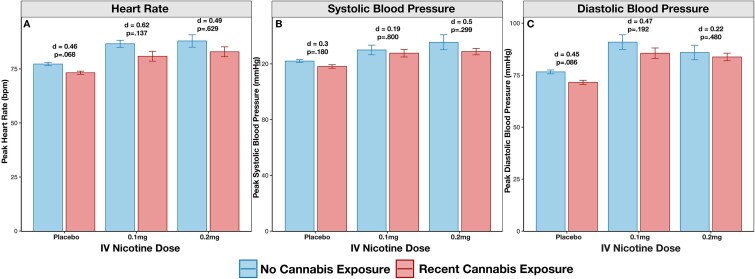
Cardiovascular effects of intravenous nicotine by cannabis exposure status. Peak values for heart rate (bpm), systolic blood pressure (mmHg), and diastolic blood pressure (mmHg) following IV nicotine administration (placebo, 0.1 mg, 0.2 mg) in individuals with recent cannabis exposure (*n* = 30) and individuals without (*n* = 30). Values represent mean ± SEM of peak responses. Cohen’s d’ effect sizes and *P*-values from post-hoc comparisons between cannabis groups are shown for each dose. No significant dose x cannabis exposure interactions were observed for any cardiovascular measure (all *P* > .50), indicating that cannabis exposure status did not modify the physiological response to nicotine. The dose-dependent increases in all cardiovascular parameters were similar between groups.

All cardiovascular measures showed expected dose-dependent increases following nicotine administration (all *P* < .0001, Figure 3). Heart rate increased by approximately 9 bpm, and systolic and diastolic blood pressure each increased by approximately 14 mmHg, at the 0.2 mg dose. Cannabis exposure status did not significantly modify the cardiovascular response to nicotine (all dose x cannabis exposure interactions *P* > .50), indicating that group differences in subjective effects were not attributable to differential physiological sensitivity to nicotine. Details for all statistical analyses are provided in Table 2.

**Table 2:** Mixed-effects model results for subjective and cardiovascular outcomes.

Outcome	Effect	*P*-value	Direction of effect
Stimulatory composite	Dose	<.0001	0.2 mg > 0.1 mg > placebo
	Cannabis exposure	.058	Cannabis > non-cannabis
	Sex	.245	—
	FTND	.0009	Higher FTND → lower response
	Dose x cannabis	.249	—
Pleasurable composite	Dose	<.0001	0.2 mg > 0.1 mg > placebo
	Cannabis exposure	.105	Cannabis > non-cannabis
	Sex	.154	—
	FTND	.0008	Higher FTND → lower response
	Dose x cannabis	.088	—
Aversive composite	Dose	<.0001	0.2 mg > 0.1 mg > placebo
	Cannabis exposure	.036	Cannabis > non-cannabis
	Sex	.033	Males > females
	FTND	.039	Higher FTND → lower response
	Dose x cannabis	.001	Individuals with cannabis exposure show greater dose-dependent increase
Heart rate	Dose	<.0001	0.2 mg > 0.1 mg > placebo
	Cannabis exposure	.132	—
	Sex	.09	—
	FTND	.511	—
	Dose x cannabis	.532	—
Systolic blood pressure	Dose	<.0001	0.2 mg > 0.1 mg > placebo
	Cannabis exposure	.317	—
	Sex	.049	Males > Females
	FTND	.85	—
	Dose x cannabis	.628	—
Nicotine self-administration	Dose	.667	—
	Cannabis exposure	.812	—
	Sex	.686	—
	FTND	.339	—
	Dose x cannabis	.319	—
Diastolic blood pressure	Dose	<.0001	0.2 mg > 0.1 mg > placebo
	Cannabis exposure	.131	—
	Sex	.9	—
	FTND	.47	—
	Dose x cannabis	.819	—

All models analyzed peak values and included dose (placebo, 0.1 mg, 0.2 mg), cannabis exposure status, sex, FTND score as covariates, and dose x cannabis exposure interaction. Subject was included as random intercept.

## Discussion

Leveraging pooled data from studies employing a double-blind IV nicotine paradigm, we found that recent, non-daily cannabis exposure was associated with greater, dose-dependent aversive responses to nicotine, evidenced by a significant dose x cannabis exposure interaction and a large between-group effect at the higher nicotine dose, while stimulatory and pleasurable responses were directionally higher, yet not statistically significant. Consistent with this, cannabis exposure status was not associated with increased nicotine self-administration behavior, and individuals with cannabis exposure exhibited a distinct clinical profile characterized by lower nicotine dependence and reduced daily cigarette consumption. Cardiovascular responses increased with dose similarly across groups.

These findings present an apparent conundrum: individuals with cannabis exposure demonstrated heightened sensitivity to nicotine’s subjective effects yet exhibited lower nicotine dependence, reduced cigarette consumption, and no increase in nicotine self-administration. Notably, neither group demonstrated a statistically significant preference for nicotine over saline in the forced-choice paradigm, which limits inferences about how the observed aversive sensitivity translates to real-world nicotine-seeking behavior; the mechanistic implications regarding reinforcement should therefore be considered preliminary. This dissociation may partly reflect the different constructs captured by each measure: the DEQ indexes moment-to-moment subjective experience, whereas the forced-choice paradigm indexes operant behavior over a constrained trial structure—and heightened subjective aversion may not immediately alter choice allocation within the limited number of trials available. Moreover, the forced-choice procedure, in which participants select between 2 pre-sampled infusions under controlled laboratory conditions, may not fully detect reinforcement differences that would emerge under more naturalistic self-administration conditions, where factors such as ad libitum dosing, social context, and the availability of alternative reinforcers influence consumption patterns. With these caveats, the reward-aversion balance framework may help contextualize these results. The reinforcing effects of drugs of abuse, including nicotine, are believed to reflect a balance between their rewarding and aversive effects.^49^ While individuals with cannabis exposure showed trends toward enhanced stimulatory and pleasurable responses to nicotine, they reported significantly and disproportionately greater aversive effects. This pattern suggests that cannabis exposure may shift the balance toward reduced net reward with amplified aversion outweighing the potentiation of reward. An alternative, non-mutually exclusive explanation involves reward contrast: if cannabis produces a qualitatively more intense rewarding experience, the relative reinforcing value of nicotine may be diminished by comparison, consistent with behavioral economic frameworks in which the availability of a more potent reinforcer devalues alternative reinforcers.

A combination of distinct mechanisms underlies the aversive and rewarding effects of nicotine. For example, α5-subtype nAChR play a crucial role in mediating the aversive effects of nicotine.^29^^,^^32^ As demonstrated in multiple studies, a genetic variation that reduces the function of α5 nAChR is associated with higher nicotine intake, presumably due to reduced aversion to nicotine.^32^^,^^50-52^ In contrast, the α4β2-type nAChR mediates the rewarding effects of nicotine.^29^^,^^31^^,^^53^ Our finding that cannabis exposure is more strongly associated with nicotine aversion than pleasurable effects suggests that cannabis exposure may preferentially modulate α5 nAChR-mediated aversive pathways, shifting the delicate reward-aversion balance toward reduced net reinforcement. Specifically, the mechanistic basis for this differential modulation likely involves a leftward shift in the aversive limb of nicotine’s inverted-U—potentially reflecting cannabinoid–cholinergic crosstalk within the medial habenula and interpeduncular nucleus α5-nAChR pathway and CB1R-mediated regulation of aversive learning. Notably, individuals with cannabis exposure who also use nicotine have been shown to have higher availability of α4β2 nAChR in the brainstem, prefrontal cortex, and thalamus.^54^ However, this upregulation appears insufficient to overcome the enhanced aversive signaling, possibly due to concurrent alterations in α5 receptor function or downstream CB1R-nicotinic receptor crosstalk that may amplify aversive pathways disproportionately.

Several candidate mechanisms may underlie this differential modulation. Cannabis-induced shifts in endocannabinoid tone could alter tonic CB1R signaling at habenular synapses, where endocannabinoids have been shown to differentially regulate glutamatergic and GABAergic transmission through both retrograde and astrocyte-mediated pathways.^55^ Additionally, cannabinoid exposure can induce lasting epigenetic modifications—including altered DNA methylation and histone acetylation—in reward-related circuits,^56^ raising the possibility that even non-daily cannabis use may produce molecular changes that shift the threshold at which aversion-encoding circuits are recruited. However, these mechanisms remain speculative in the context of nicotinic aversion and require direct experimental testing.

Preclinical findings on the impact of exposure to phytocannabinoids on nicotine reinforcement are mixed. For instance, THC exposure in rodents enhances both the likelihood of acquiring nicotine self-administration and the reward value of nicotine.^26^ More broadly, CB1R agonists have produced bidirectional effects on nicotine reinforcement depending on dose, schedule, and timing: for example, the CB1R agonist WIN55,212-2 increases nicotine self-administration under progressive-ratio schedules and precipitates reinstatement of nicotine seeking—effects prevented by CB1R blockade.^57^ Cannabidiol (CBD) is a pleiotropic phytocannabinoid that acts as a negative allosteric modulator at CB1R, antagonist at G protein–coupled receptor 55 (GPR55),^58^^,^^59^ agonist/desensitizer at transient receptor potential vanilloid 1 (TRPV1),^60^ and functional 5-hydroxytryptamine 1A (5-HT1A) receptor agonist and can indirectly elevate anandamide by inhibiting uptake/fatty acid amide hydrolase (FAAH).^61^ Recent work showed that CBD decreases nicotine self-administration and attenuates withdrawal in mice.^62^ Mechanistically, CBD’s attenuation of CB1R signaling and engagement of 5-HT1A/TRPV1 pathways could dampen mesolimbic dopamine responses to nicotine, reduce cue-driven incentive salience, and blunt withdrawal-related negative affect.^63^ Notably, THC has also been reported to decrease nicotine self-administration in rats under certain conditions.^64^ Together, these findings underscore the complex, bidirectional cannabinoid–nicotinic interactions that hinge on cannabinoid ligand, receptor target, dose, reinforcement schedule, and the timing and context of administration.

Limited clinical evidence examining cannabis-nicotine interactions has yielded seemingly contradictory results that, when considered together, reveal potential distinctions between acute and chronic effects. Penetar et al. administered transdermal nicotine patches (21 mg) to individuals who use cannabis and found that nicotine pretreatment enhanced subjective responses to smoked cannabis, increasing particularly ratings of stimulation and euphoria.^40^ In contrast, Peters et al. found that acute cannabis administration did not alter subsequent tobacco smoking patterns—specifically, the number of puffs taken, puff duration, inter-puff intervals, or total smoke exposure—despite producing robust cannabis intoxication.^41^ Consistent with this, population-based studies have found that average daily cigarette consumption does not differ between individuals who co-use cannabis and nicotine and individuals who smoke tobacco cigarettes only.^65^^,^^66^

### Limitations

This study has several methodological strengths: The use of IV nicotine administration provided precise dosing control and eliminated confounding from tobacco smoke constituents, allowing isolation of nicotine’s pharmacodynamic effects. The within-subjects, placebo-controlled design enhanced statistical power despite the modest sample size. The inclusion of both subjective and objective measures, along with comprehensive baseline characterization, strengthened our ability to detect cannabis-related differences.

However, several limitations warrant consideration. First, cannabis exposure was classified via self-report and baseline THC-COOH only; we lacked fine-grained characterization of use patterns (recency, frequency/duration, route, product type, THC potency, THC:CBD ratios, age of initiation) or synergistic effects of concurrent use. Given preclinical evidence that THC and CBD can exert divergent effects on nicotine reinforcement,^62^^,^^64^ our inability to distinguish THC-dominant from CBD-rich exposure may have obscured meaningful subgroups and precluded dose–response modeling. Because cannabis status was not randomized, the between-group comparison is observational, limiting causal inference about whether altered nicotine sensitivity precedes or follows cannabis exposure. Consequently, we cannot determine whether the observed effects in participants with recent cannabis exposure reflect acute residual effects, short-term cannabis-induced neuroadaptations, or pre-existing differences in nicotine sensitivity that preceded cannabis initiation. Power was optimized for large effects; larger samples will be needed to confirm the observed trends in stimulatory and pleasurable responses. Residual confounding is possible given baseline differences in smoking heaviness and dependence (eg, CPD, FTND, age), and pooling across 2 parent studies may introduce unmodeled study-level heterogeneity if not explicitly accounted for; sensitivity analyses adjusting for parent study, age, CPD, and race/ethnicity confirmed that the dose x cannabis exposure interaction for aversive effects remained significant across all specifications (Supplementary Tables S1-S5). CO cutoffs used to verify overnight abstinence (≤8-10 ppm across parent studies) are consistent with the IV nicotine literature for this purpose, though they are higher than the most stringent thresholds developed to confirm 24-hour smoking cessation.^67^ Still, cutoffs were applied uniformly regardless of cannabis exposure status. Finally, generalizability is limited to adults who use tobacco cigarettes with recent, non-daily cannabis exposure; results may not extend to those who use cannabis daily, individuals with cannabis use disorder, or those who exclusively use electronic cigarettes. Concurrent use of other nicotine/tobacco products (eg, e-cigarettes, cigars, smokeless tobacco) was not systematically assessed and may represent an unmeasured confounder. Baseline plasma nicotine and cotinine concentrations stratified by cannabis exposure status were not available for the combined sample, precluding assessment of whether residual nicotine levels differed between groups.

### Future directions

The rapidly evolving cannabis policy landscape, with increasing legalization and rising THC potency in commercial products, necessitates renewed attention to cannabis-tobacco interactions. As cannabis use becomes more prevalent and products become more potent, the population of individuals with altered nicotine sensitivity may expand. This could have unintended consequences for public health efforts to reduce tobacco use, particularly as both substances are often initiated during adolescence when the brain is particularly vulnerable to drug-induced neuroadaptations. Our findings underscore the need for tailored interventions that account for the altered pharmacodynamic profile of nicotine in individuals who use cannabis.

Several avenues warrant future investigation. Longitudinal studies tracking nicotine sensitivity before and after cannabis initiation could establish temporal relationships and identify critical periods of vulnerability. Neuroimaging studies combining IV nicotine challenges with brain positron emission tomography (PET) and functional magnetic resonance imaging (fMRI) could elucidate the neural circuits underlying enhanced aversive responses in individuals with cannabis exposure. Investigation of sex differences is particularly important given evidence for sex differences in cannabinoid-nicotine interactions.^40^ Mechanistic studies should test whether the observed aversive amplification reflects cannabis-induced alterations in endocannabinoid signaling within the MHb–IPN^55^, epigenetic regulation of genes encoding nicotinic receptor subunits,^56^ or changes in α5-containing nAChR expression or function. Pharmacogenomic approaches^68^ examining whether the CHRNA5 rs16969968 variant—which reduces α5 function and increases nicotine intake^32^—moderates the cannabis x nicotine interaction could directly test whether the effect operates through α5 aversion signaling. Combining IV nicotine challenges with PET imaging using α4β2-selective radioligands and fMRI could determine whether individuals who use cannabis exhibit differential habenular activation at higher nicotine doses. The roles of non-CB1 mechanisms, including CB2 receptors, TRPV1 channels, and PPARα, also warrant investigation. Additionally, investigating how different cannabinoids (THC vs CBD) and routes of administration (smoking vs vaping vs edibles) influence nicotine responses could inform more nuanced clinical recommendations. The contrasting effects of THC and CBD on nicotine reinforcement observed in preclinical studies^62^^,^^64^ suggest that cannabinoid profile may be a critical determinant of cannabis-nicotine interactions. Future studies should prospectively characterize cannabis use patterns, including frequency, duration, product type, THC potency, THC:CBD ratio, route of administration, and age of initiation, to enable dose–response modeling of cannabis exposure effects on nicotine pharmacodynamics.

## Conclusions

Using a rigorously controlled IV nicotine paradigm, we demonstrate that recent non-daily cannabis exposure selectively amplifies nicotine’s aversive effects at clinically relevant doses, with no evidence of altered cardiovascular reactivity or increased nicotine choice. The dissociation between selectively heightened aversive responding and unchanged reinforcement highlights the complex, multifaceted nature of cannabis–nicotine interactions and indicates that shifts in subjective domains do not necessarily translate into greater primary reinforcement in humans. These findings underscore the need for tailored interventions that account for potential alterations in nicotine’s pharmacodynamic profile among individuals with cannabis exposure. As legalization expands and co-use patterns evolve, clarifying these interactions will be increasingly important for developing effective tobacco-control strategies. Future research should elucidate underlying mechanisms (eg, cannabinoid–cholinergic cross-talk and dose–response context) and translate them into targeted prevention and cessation approaches.

## Supplementary Material

Supplementary_material_pyag030

## Data Availability

De-identified individual participant data underlying the results reported in this article (including the analysis dataset and variable dictionary), the analysis code, and study materials will be made available by the corresponding author upon reasonable request for non-commercial academic purposes.

## Notes

Author Contributions: Drs M. Sofuoglu and J. P. De Aquino contributed equally and share senior authorship.
