# Supplementary material for: Recent cannabis exposure and acute nicotine effects: insights from randomized, double-blind, placebo-controlled intravenous nicotine studies
Source: Int J Neuropsychopharmacol. 2026 May 26;29(7):pyag030. doi: 10.1093/ijnp/pyag030 (PMC13322297; doi:10.1093/ijnp/pyag030)
Supplement: Supplementary_material_pyag030 [file supplementary_material_pyag030.zip › Supplement_pyag030.docx]

**Supplementary Figure S1. Participant flow and crossover session schema for pooled IV nicotine studies (N=60).**


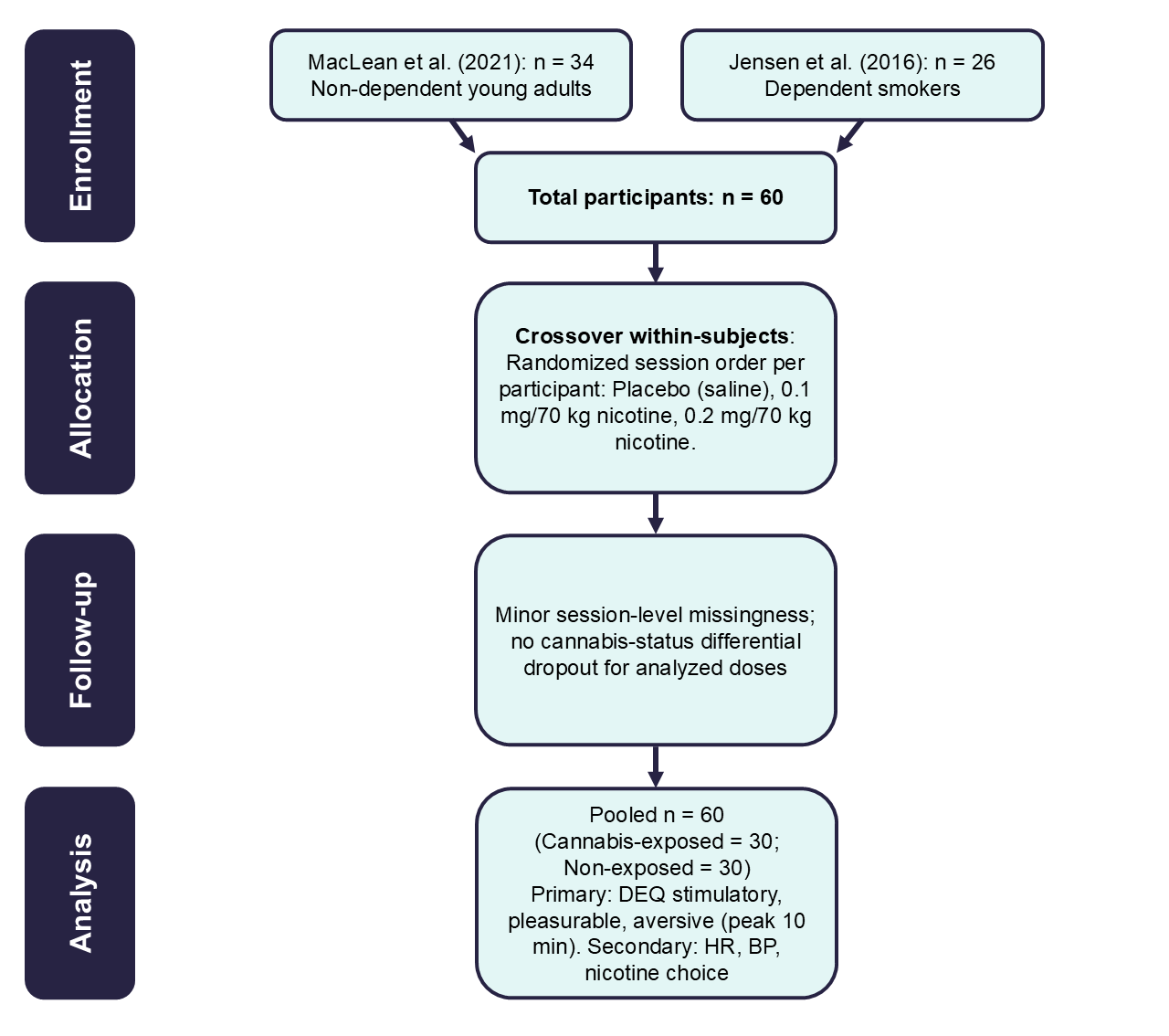


Participants from two parent studies (n=60) completed randomized sessions with placebo, 0.1 mg/70 kg, and 0.2 mg/70 kg IV nicotine; minor session-level missingness occurred without cannabis-status differential dropout. Primary outcomes were DEQ stimulatory, pleasurable, and aversive ratings (peak 10 min); secondary: heart rate, blood pressure, and nicotine choice.

**Sensitivity Analyses: Alternative Covariate Specifications**

To evaluate the robustness of our primary findings to alternative covariate specifications, we re-estimated all models with five additional configurations: (1) age, (2) cigarettes per day (CPD), (3) race/ethnicity, (4) parent study, and (5) body weight (kg) as fixed effects, each added to the primary model (sex + FTND).

**Supplementary Table S1. Type III ANOVA p-values for DEQ Composites Under Alternative Covariate Specifications**

| **Outcome** | **Effect** | **+ Age** | **+ CPD** | **+ Race** | **+ Parent Study** | **+ Weight** |
| --- | --- | --- | --- | --- | --- | --- |
| Stimulatory | Dose | <.001 | <.001 | <.001 | <.001 | <.001 |
|  | Cannabis | .142 | .087 | .048 | .488 | .117 |
|  | Dose x Cannabis | .273 | .243 | .250 | .279 | .198 |
|  | FTND | .021 | .159 | .007 | .976 | .002 |
|  | Sex | .371 | .229 | .230 | .815 | .233 |
| Pleasurable | Dose | <.001 | <.001 | <.001 | <.001 | <.001 |
|  | Cannabis | .271 | .160 | .102 | .692 | .179 |
|  | Dose x Cannabis | .098 | .083 | .087 | .092 | .089 |
|  | FTND | .031 | .240 | .003 | .960 | .003 |
|  | Sex | .263 | .132 | .165 | .567 | .109 |
| Aversive | Dose | <.001 | <.001 | <.001 | <.001 | <.001 |
|  | Cannabis | .090 | .051 | .032 | .245 | .079 |
|  | Dose x Cannabis | .002 | .001 | .001 | .001 | .003 |
|  | FTND | .251 | .512 | .098 | .609 | .042 |
|  | Sex | .056 | .029 | .028 | .129 | .056 |

*Note.* Each sensitivity model adds the listed covariate to the primary specification (sex + FTND). All models used log-transformed peak composites with participant as a random intercept and included dose, cannabis exposure status, sex, FTND score, and the dose x cannabis exposure interaction.

**Supplementary Table S2. Aversive DEQ Pairwise Comparisons (Cannabis vs. Non-Cannabis) by Nicotine Dose Under Alternative Covariate Specifications**

| **Model** | **Placebo: diff (p) [d’]** | **0.1 mg/70kg: diff (p) [d’]** | **0.2 mg/70kg: diff (p) [d’]** |
| --- | --- | --- | --- |
| + Age | 0.1 (.771) [0.12] | 0.4 (.243) [0.53] | 1.0 (.007) [1.24] |
| + CPD | 0.2 (.624) [0.20] | 0.5 (.168) [0.63] | 1.1 (.003) [1.34] |
| + Race | 0.2 (.490) [0.28] | 0.6 (.124) [0.71] | 1.2 (.002) [1.42] |
| + Parent Study | -0.1 (.783) [0.10] | 0.3 (.486) [0.30] | 0.9 (.019) [1.03] |
| + Weight | 0.2 (.624) [0.22] | 0.6 (.182) [0.68] | 1.1 (.009) [1.33] |

*Note.* diff = estimated marginal mean difference (Cannabis minus Non-cannabis) on the log scale; values in brackets are Cohen's d' effect sizes, with positive values indicating higher ratings in the cannabis-exposed group.

**Supplementary Table S3. Correlation Matrix of Covariates and Group Variables (N = 60)**

|  | **Cannabis** | **Study** | **Age** | **Sex** | **FTND** | **CPD** | **Weight** |
| --- | --- | --- | --- | --- | --- | --- | --- |
| Cannabis | 1.00 | .40 | -.35 | .17 | -.29 | -.26 | -.30 |
| Study | .40 | 1.00 | -.69 | .22 | -.62 | -.72 | -.35 |
| Age | -.35 | -.69 | 1.00 | -.17 | .44 | .59 | .37 |
| Sex | .17 | .22 | -.17 | 1.00 | -.04 | -.04 | .13 |
| FTND | -.29 | -.62 | .44 | -.04 | 1.00 | .63 | .31 |
| CPD | -.26 | -.72 | .59 | -.04 | .63 | 1.00 | .31 |
| Weight | -.30 | -.35 | .37 | .13 | .31 | .31 | 1.00 |

*Note.* Pearson correlations (point-biserial for binary variables). Cannabis: 1 = recent use; Study: 1 = Study 2; Sex: 1 = Male. Parent study was strongly correlated with FTND (r = -.62), CPD (r = -.72), and age (r = -.69), reflecting systematic differences in eligibility criteria across studies. Cannabis use status and parent study were also correlated (r = .40): 26.9% of Study 1 participants had recent cannabis exposure compared to 67.6% in Study 2. Body weight was moderately correlated with cannabis use status (r = -.30), with participants reporting recent cannabis use weighing less on average.

**Supplementary Table S4. Cardiovascular ANOVA p-values Under Alternative Covariate Specifications**

| **Outcome** | **Effect** | **+ Age** | **+ CPD** | **+ Race** | **+ Parent Study** | **+ Weight** |
| --- | --- | --- | --- | --- | --- | --- |
| Systolic BP | Dose | <.001 | <.001 | <.001 | <.001 | <.001 |
|  | Cannabis | .663 | .416 | .371 | .865 | .574 |
|  | Dose x Cannabis | .673 | .626 | .623 | .657 | .725 |
|  | FTND | .202 | .205 | .307 | .035 | .355 |
|  | Sex | .007 | .039 | .037 | .003 | .198 |
| Diastolic BP | Dose | <.001 | <.001 | <.001 | <.001 | <.001 |
|  | Cannabis | .357 | .196 | .150 | .593 | .305 |
|  | Dose x Cannabis | .816 | .831 | .824 | .847 | .900 |
|  | FTND | .106 | .034 | .219 | .005 | .887 |
|  | Sex | .471 | .891 | .983 | .277 | .685 |
| Heart Rate | Dose | <.001 | <.001 | <.001 | <.001 | <.001 |
|  | Cannabis | .092 | .139 | .114 | .094 | .613 |
|  | Dose x Cannabis | .525 | .533 | .529 | .523 | .335 |
|  | FTND | .848 | .509 | .327 | .972 | .233 |
|  | Sex | .065 | .090 | .082 | .066 | .126 |

*Note.* Peak values per session (untransformed). No cannabis main effects or dose x cannabis interactions reached significance in any specification.

**Supplementary Table S5. Nicotine Self-Administration ANOVA p-values Under Alternative Covariate Specifications**

| **Effect** | **+ Age** | **+ Race** | **+ Paradigm/Study** | **+ Weight** |
| --- | --- | --- | --- | --- |
| Dose | .666 | .585 | .667 | .460 |
| Cannabis | .825 | .996 | .813 | .638 |
| Dose x Cannabis | .319 | .353 | .319 | .158 |
| FTND | .366 | .482 | .443 | .275 |
| Sex | .693 | .533 | .687 | .647 |

*Note.* Paradigm/Study reflects the 6-choice (Study 1) vs. 10-choice (Study 2) trial structure. CPD was not included as a sensitivity covariate for self-administration because FTND and CPD are highly correlated (r = .63). No effects reached significance in any specification.

**Individual DEQ Item Analyses**

To examine which individual items contributed most to the composite-level findings, we repeated the primary analysis for each of the nine DEQ items comprising the three composite scores.

**Supplementary Table S6. DEQ Individual Item Analyses**

***Panel A. Type III ANOVA p-values***

| **DEQ Item** | **Composite** | **Dose** | **Cannabis** | **Sex** | **FTND** | **Dose x Cannabis** |
| --- | --- | --- | --- | --- | --- | --- |
| Stimulated | Stimulatory | <.001* | .018* | .513 | .005* | .153 |
| High | Stimulatory | <.001* | .035* | .169 | .006* | .312 |
| Drug Strength | Stimulatory | <.001* | .131 | .219 | <.001* | .329 |
|  |  |  |  |  |  |  |
| Good Drug Effects | Pleasurable | <.001* | .088 | .204 | .002* | .025* |
| Like Drug Effects | Pleasurable | <.001* | .074 | .205 | .001* | .237 |
| Want More | Pleasurable | <.001* | .145 | .085 | .001* | .147 |
|  |  |  |  |  |  |  |
| Anxious | Aversive | <.001* | .090 | .028* | .033* | .047* |
| Down | Aversive | <.001* | .014* | .200 | .180 | <.001* |
| Bad Drug Effects | Aversive | <.001* | .214 | .091 | .048* | .017* |

* *p* < .05. Items grouped by composite membership.

***Panel B. Pairwise Effect Sizes (Cohen's d'): Cannabis Exposure vs. No Cannabis Exposure at Each Dose***

| **DEQ Item** | **Composite** | **Placebo d' (p)** | **0.1 mg/70 kg d' (p)** | **0.2 mg/70 kg d' (p)** |
| --- | --- | --- | --- | --- |
| Stimulated | Stimulatory | 0.51 (.165) | 0.96 (.025)* | 1.05 (.014)* |
| High | Stimulatory | 0.63 (.141) | 0.87 (.067) | 1.09 (.022)* |
| Drug Strength | Stimulatory | 0.28 (.445) | 0.66 (.120) | 0.66 (.121) |
|  |  |  |  |  |
| Good Drug Effects | Pleasurable | 0.27 (.533) | 0.82 (.089) | 1.06 (.029)* |
| Like Drug Effects | Pleasurable | 0.42 (.305) | 0.89 (.055) | 0.81 (.080) |
| Want More | Pleasurable | 0.31 (.495) | 0.76 (.128) | 0.84 (.091) |
|  |  |  |  |  |
| Anxious | Aversive | 0.18 (.623) | 0.80 (.061) | 0.82 (.055) |
| Down | Aversive | 0.50 (.300) | 1.24 (.020)* | 1.71 (.002)* |
| Bad Drug Effects | Aversive | 0.07 (.847) | 0.34 (.441) | 0.97 (.028)* |

* *p* < .05. Positive *d*' values indicate individuals with recent cannabis exposure reported higher ratings than those without.

***Panel C. FTND Coefficients***

| **DEQ Item** | **Composite** | **β** | **SE** | ***p*** |
| --- | --- | --- | --- | --- |
| Stimulated | Stimulatory | -0.392 | 0.140 | .007* |
| High | Stimulatory | -0.391 | 0.143 | .008* |
| Drug Strength | Stimulatory | -0.496 | 0.140 | <.001* |
|  |  |  |  |  |
| Good Drug Effects | Pleasurable | -0.455 | 0.148 | .003* |
| Like Drug Effects | Pleasurable | -0.469 | 0.147 | .002* |
| Want More | Pleasurable | -0.499 | 0.152 | .002* |
|  |  |  |  |  |
| Anxious | Aversive | -0.258 | 0.121 | .037* |
| Down | Aversive | -0.169 | 0.126 | .185 |
| Bad Drug Effects | Aversive | -0.257 | 0.130 | .052 |

* *p* < .05. β = standardized coefficient for scaled FTND score. Negative values indicate higher nicotine dependence predicted lower subjective ratings.

Individual item analyses were consistent with the composite-level findings reported in the main text. For the **aversive composite**, the dose x cannabis interaction was driven most strongly by "feeling down" (*p* < .001), which showed the largest pairwise effect size in the study (*d*' = 1.71 at 0.2 mg/70 kg, *p* = .002), followed by "bad drug effects" (interaction *p* = .017) and "anxious" (interaction *p* = .047). All three aversive items showed the same pattern: minimal group differences at placebo with progressively larger differences at active doses, consistent with a dose-dependent moderation of aversive nicotine sensitivity by cannabis use status.

For the **stimulatory composite**, individuals with recent cannabis exposure reported significantly higher ratings of "stimulated" (cannabis main effect *p* = .018) and "high" (*p* = .035), with significant pairwise differences emerging at active doses (stimulated: *d*' = 0.96 to 1.05; high: *d*' = 1.09 at 0.2 mg). "Drug strength" showed the same directional pattern but did not reach significance for the cannabis main effect (*p* = .131), suggesting the composite-level trend (*p* = .058) was primarily driven by "stimulated" and "high." None of the stimulatory items showed a significant dose x cannabis interaction, indicating that cannabis-related differences in stimulatory effects were relatively consistent across dose levels.

For the **pleasurable composite**, "good drug effects" was the only individual item showing a significant dose x cannabis interaction (*p* = .025), with pairwise differences reaching significance at 0.2 mg/70 kg (*d*' = 1.06, *p* = .029). "Like drug effects" and "want more" showed medium-to-large effect sizes at active doses (*d*' = 0.76 to 0.89) but did not reach statistical significance, likely reflecting insufficient power for individual item comparisons.

FTND was a significant predictor for 7 of 9 items, with higher dependence scores consistently predicting attenuated subjective responses (Panel C). Notably, the two items for which FTND was not significant — "feeling down" (*p* = .185) and "bad drug effects" (*p* = .052) — were the same items that most strongly drove the dose x cannabis interaction. This dissociation suggests that cannabis-related differences in aversive nicotine sensitivity are not merely a proxy for nicotine dependence severity, as the items most sensitive to cannabis status were those least explained by FTND.
